# Editorial: Dietary patterns and oxidative stress: implications for obesity, T2D, and cancer management

**DOI:** 10.3389/fnut.2025.1758019

**Published:** 2026-01-12

**Authors:** Qingyu Wang, Yixuan Li, Dongxian Guan

**Affiliations:** 1 Medical Research Center, Beijing Chaoyang Hospital, Capital Medical University, Beijing, China; 2 Key Laboratory of Functional Dairy, Department of Nutrition and Health, China Agricultural University, Beijing, China; 3 Institute of Modern Biology, Nanjing University, Nanjing, China

**Keywords:** calorie restriction, cancer, high calorie, metabolic disease, ROS

Understanding how dietary patterns modulate internal oxidative stress is becoming increasingly important for explaining the rising global burden of metabolic diseases, including obesity, type 2 diabetes (T2D), and cancer. Excessive caloric intake has been shown to promote the generation of reactive oxygen species (ROS), which in turn drive chronic inflammation, metabolic dysfunction, and oxidative cellular injury. Thus, high-calorie dietary patterns and the oxidative stress they induce contribute to numerous pathological processes, including tumor initiation and progression (1, 2), insulin resistance (3), β-cell dysfunction (4), and obesity-related tissue impairment (5). However, evidence regarding which specific dietary components and molecular pathways exacerbate or alleviate oxidative injury remains limited (6). To address this gap, this Research Topic presents studies that elucidate the mechanistic links between dietary exposures, oxidative stress, inflammation, and chronic disease risk, while also highlighting emerging biomarkers and potential intervention strategies.

Calorie excess, nutrient imbalance, and Western dietary habits elevate the production of ROS. Two complementary studies by Han et al. and Ying et al. examine the impact of diet-derived inflammatory and oxidative indices on mortality risk. Han et al. demonstrate that higher Dietary Inflammatory Index scores are significantly associated with increased all-cause and cardiovascular mortality in non-diabetic adults, whereas higher Dietary Oxidative Balance Scores are associated with lower mortality risk. Ying et al. extend these insights to overweight and obese populations and report that elevated oxidative balance scores correlate with reduced all-cause and cardiovascular mortality in these groups. A global perspective is provided by Hou et al., who quantify the burden of T2D-related chronic kidney disease (CKD) attributable to dietary risks using data from the Global Burden of Disease (GBD) 2021 study. Their analysis shows that diets low in fruits, whole grains, and vegetables and diets high in processed meats are major contributors to the burden of T2D-related CKD. Taken together, these findings highlight the value of dietary indices that capture inflammatory and oxidative potential as tools for predicting chronic disease risk.

It has become increasingly clear that our diets influence far more than caloric intake. The interplay between micronutrients and metabolic homeostasis is highlighted by Lin et al., who report that the risks of overweight and obesity are inversely associated with serum iron levels, transferrin saturation, and dietary iron intake. Their findings suggest that iron status may shape energy metabolism, adipocyte function, and inflammatory processes. Dietary intake also modulates gut microbiome composition, and both probiotics and prebiotics have attracted considerable attention. Niu et al. demonstrate that a pre-screened synbiotic combination containing *Bifidobacterium animalis* subsp. *lactis* MN-Gup, galacto-oligosaccharides, and xylo-oligosaccharides reduce body fat, LDL cholesterol, and waist circumference, while promoting beneficial gut bacteria and bile acid profiles. Sun et al. show that heat-killed *B. longum* BBMN68 combined with inulin improves lipid metabolism and increases short-chain fatty acids in high-fat diet–induced obese rats, supporting a post-biotic approach to oxidative and metabolic regulation. Collectively, these interventions emphasize that microbiota-directed therapies might modulate oxidative stress indirectly through intestinal metabolites, bile acid signaling, and the maintenance of gut barrier integrity.

High oxidative status has long been recognized as a contributing factor in cancer progression. An et al. demonstrate that low tumor expression of key antioxidant enzymes in colorectal cancer, particularly SOD and PRX4, is associated with distant metastasis, systemic inflammation, poor differentiation, and reduced survival. These findings reinforce the concept that diminished endogenous antioxidant capacity may accelerate tumor progression. This work also highlights the potential value of tissue-based antioxidant markers as prognostic tools that could complement existing clinical assessments.

Overall, the studies in this Research Topic advance our understanding of how dietary patterns, micronutrient status, gut microbiota, and inflammatory burden intersect with oxidative stress to influence the development and progression of obesity, T2D, and cancer. They also strengthen the evidence that oxidative stress is not just a downstream consequence of poor metabolic health but also an important mediator linking dietary exposures with chronic disease outcomes.
